# Strep A: challenges, opportunities, vaccine-based solutions and economics

**DOI:** 10.1038/s41541-024-00864-6

**Published:** 2024-04-19

**Authors:** David E. Bloom, Richard W. Titball, Jonathan Carapetis

**Affiliations:** 1 Harvard T.H. Chan School of Public Health, Boston, MA USA; 2 Department of Biosciences, University of Exeter, Exeter, UK; 3 Wesfarmers Centre for Vaccines and Infectious Diseases, Telethon Kids Institute, University of Western Australia, Perth, WA Australia; 4 Perth Children’s Hospital, Perth, WA Australia

*Streptococcus pyogenes* (Strep A) is a leading cause of morbidity and mortality across the globe, annually causing hundreds of millions of cases of disease. The spectrum of disease ranges from relatively mild infections such as pharyngitis and impetigo to invasive and life-threatening conditions such as necrotizing fasciitis, sepsis, scarlet fever, glomerulonephritis, acute rheumatic fever, and rheumatic heart disease. Although antibiotics can be used to treat Strep A disease, they are often unable to prevent the development of serious forms of disease and it is unclear how they would be used at scale without contributing to drug resistance, especially among bystander pathogens. Therefore, there is an urgent need to identify ways of combatting this pathogen more effectively. This *NPJ Vaccines* collection, entitled “Special Collection: Streptococcus A vaccines,” investigates the value proposition for Strep A vaccines. The collection provides a platform for experts and stakeholders to inform the readership of ongoing research to advance Strep A vaccine development and implementation, in order to widen and deepen our understanding of the value of vaccination.

Momentum for a Strep A vaccine is finally building: the World Health Organization declared it a priority in 2014, as did the Product Development for Vaccines Advisory Committee in 2016. WHO published an R&D roadmap for Strep A vaccine development in 2018. The following year, the Strep A Vaccine Global Consortium (SAVAC) was established. Through a network of specialists in epidemiology, health economics, medicine, immunology, and global health policy, SAVAC’s mission is to “ensure that safe, effective and affordable Strep A vaccines are available and implemented to decrease the burden of Strep A disease in the most in need”.

The collection is centred around the proposal that prospective Strep A vaccines have been substantially undervalued. This undervaluation stems in part from a reliance on methodologies for health technology assessment that adopt a narrow health payer-centric view of vaccination benefits. This approach neglects many health, economic, and social benefits, leading directly to underinvestment in vaccine development and coverage. This underinvestment is of particular impact in low- and middle-income countries, where many of the most serious forms of Strep A disease occur.

Reforming the health ecosystem to value and appropriately utilize vaccinations will ultimately require everything from reform of our institutions and practices for vaccine regulation; to advances in the way we conceptualize and operationalize health technology assessment; to increased investments in vaccine development, testing, and delivery. This means adopting a broad societal perspective that requires an appropriately meaningful valuation of the health, economic, and social burdens of diseases. Achieving the goals of this workstream will require the consideration of three sets of issues that are fundamental to undertaking meaningful assessments of value: perspective, sources of value, and metrics.

We hope that you find this collection both valuable and thought provoking. Importantly, the approaches outlined in this collection might also find utility for the assessment of the value proposition for vaccines against other infectious diseases.

